# Low genetic diversity of *Plasmodium falciparum* merozoite surface protein 1 and 2 and multiplicity of infections in western Ethiopia following effective malaria interventions

**DOI:** 10.1186/s12936-022-04394-1

**Published:** 2022-12-15

**Authors:** Geletta Tadele, Fatou K. Jaiteh, Mary Oboh, Eniyou Oriero, Sisay Dugassa, Alfred Amambua-Ngwa, Lemu Golassa

**Affiliations:** 1 Aklilu Lemma Institute of Pathobiology, Addis Ababa University, Addis Ababa, Ethiopia; 2 Medical Research Council Unit the Gambia, London School of Hygiene and Tropical Medicine, Serrekunda, The Gambia

**Keywords:** MOI, Genetic diversity, *P. falciparum*, Malaria transmission, Western Ethiopia

## Abstract

**Background:**

Genetic diversity of malaria parasites can inform the intensity of transmission and poses a major threat to malaria control and elimination interventions. Characterization of the genetic diversity would provide essential information about the ongoing control efforts. This study aimed to explore allelic polymorphism of merozoite surface protein 1 (*msp1*) and merozoite surface protein 2 (*msp2*) to determine the genetic diversity and multiplicity of *Plasmodium falciparum* infections circulating in high and low transmission sites in western Ethiopia.

**Methods:**

Parasite genomic DNA was extracted from a total of 225 dried blood spots collected from confirmed uncomplicated *P. falciparum* malaria-infected patients in western Ethiopia. Of these, 72.4% (163/225) and 27.6% (62/225) of the samples were collected in high and low transmission areas, respectively. Polymorphic *msp1* and *msp2* genes were used to explore the genetic diversity and multiplicity of falciparum malaria infections. Genotyping of *msp1* was successful in 86.5% (141/163) and 88.7% (55/62) samples collected from high and low transmission areas, respectively. Genotyping of *msp2* was carried out among 85.3% (139/163) and 96.8% (60/62) of the samples collected in high and low transmission sites, respectively. *Plasmodium falciparum msp1* and *msp2* genes were amplified by nested PCR and the PCR products were analysed by QIAxcel ScreenGel Software. A P-value of less or equal to 0.05 was considered significant.

**Results:**

High prevalence of falciparum malaria was identified in children less than 15 years as compared with those  ≥ 15 years old (AOR = 2.438, P = 0.005). The three allelic families of *msp1* (K1, MAD20, and RO33) and the two allelic families of *msp2* (FC27 and 3D7), were observed in samples collected in high and low transmission areas. However, MAD 20 and FC 27 alleles were the predominant allelic families in both settings. *Plasmodium falciparum* isolates circulating in western Ethiopia had low genetic diversity and mean MOI. No difference in mean MOI between high transmission sites (mean MOI 1.104) compared with low transmission area (mean MOI 1.08) (p > 0.05). The expected heterozygosity of *msp1* was slightly higher in isolates collected from high transmission sites (He = 0.17) than in those isolates from low transmission (He = 0.12). However, the heterozygosity of *msp*2 was not different in both settings (*Pfmsp2*: 0.04 in high transmission; pf*msp2*: 0.03 in low transmission).

**Conclusion:**

*Plasmodium falciparum* from clinical malaria cases in western Ethiopia has low genetic diversity and multiplicity of infection irrespective of the intensity of transmission at the site of sampling. These may be signaling the effectiveness of malaria control strategies in Ethiopia; although further studies are required to determine how specific intervention strategies and other parameters that drive the pattern.

## Background

Malaria remains a global public health problem with 229 million cases and 409,000 deaths globally in 2019. *Plasmodium falciparum* remains the most dangerous malaria parasite species to humans with important public health consequences mostly in Africa [1].

Multiple *Plasmodium* strains (also known as multiplicity of infection, MOI) are common in malaria-endemic areas, which may result from the bite of mosquitoes infected with more than one clone or from multiple bites [2]. Interaction and competition of different strains for resources within a host during their life cycles may be important for survival and promotes diversity of the parasite [3, 4]. The presence of multiple infecting strains may also affect key phenotypic traits, including drug resistance [5, 6] and risk of severe disease [7, 8]. Characterizing the genetic diversity of malaria in infected individuals and populations of different endemic settings could help determine the effectiveness of malaria interventions. A multiplicity of the parasite has been proposed as a marker to measure changes in transmission intensity in time and space [9, 10].

Malaria parasites genomic epidemiology studies have been assessed using MOI and the proportion of polyclonal infections (also termed ‘multiple clone infections’ [9, 11]. It is generally assumed that MOI is higher in high transmission settings, while in low transmission settings, most individuals might carry single clone infections. A correlation between transmission intensity and MOI has been reported [12–14]. In a study done in Eswatini showed an absence of association between a multiplicity of infection and transmission intensity, most likely due to infections being imported [15]. In Ethiopia, altitude, and climate (rainfall and temperature) are the most significant determinants of malaria transmission. *Plasmodium falciparum* is the dominant malaria parasite, accounting for 60–70% of all malaria cases [16]. Over the last two decades, malaria morbidity and mortality have been reduced dramatically in the country following intensive use of insecticide residual spray, long-lasting insecticide-treated nets, chemotherapies, improved diagnosis, and case management recent years [17]. Towards local strategies for malaria elimination; it is important to assess the genetic diversity of malaria parasites in different settings to determine the effectiveness of the ongoing interventions and to better understand the parasite population circulating in a given geographic area. In this study, we focus on Western Ethiopia, where there is relatively limited information about the genetic diversity and MOI of *P. falciparum*. This study aimed to compare the genetic diversity of *P. falciparum* isolates collected from high and low transmission sites, in western Ethiopia.

## Methods

### Study site and period

**Fig. 1:**
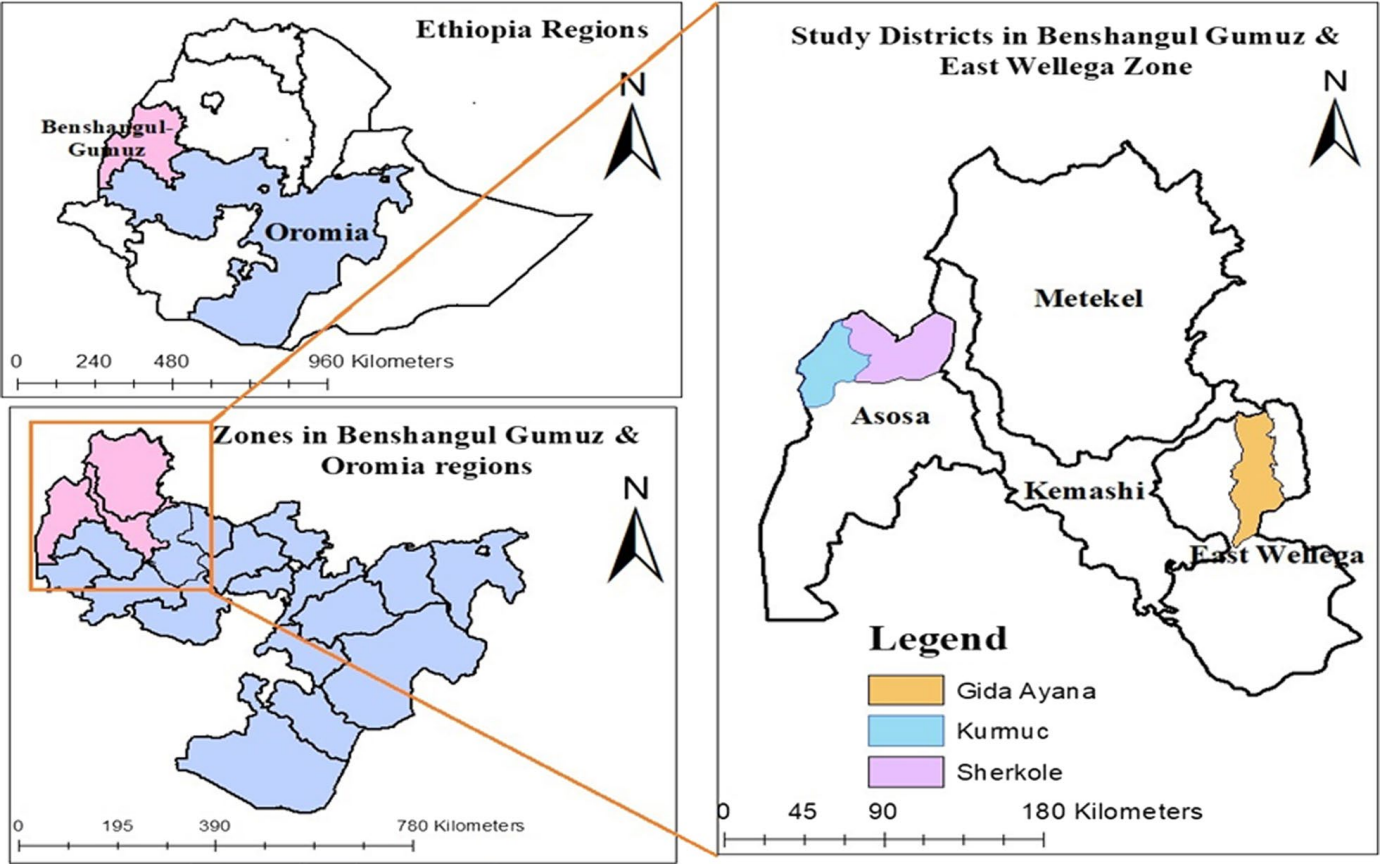
Study area map, Gida Ayana, Sherkole, and Kurmuk districts, Western Ethiopia

This study was carried out during the high malaria transmission season from September to December 2020 in four health centres located in areas with different levels of malaria transmission in the western part of Ethiopia. Western Ethiopia contains the regions of Gambela, Benishangul-Gumuz, and the western zones of Oromia. This study was conducted in two regions; Benishangul-Gumuz and Oromia. Sherkole and Kumurc districts from Benishangul-Gumaz and Gida Ayana district from the Oromia regions were selected to include four health centres for data collection (Fig. 1).

Inhabitants in Sherkole and kurmuc districts are 35,954 and 24,345, respectively. Similarly, the population size of the Gida Ayana district is 151,290. In the study districts; Sherkole (*P. falciparum* = 12, 284 and *P. vivax* = 74); Kurmuc (*P. falciparum* = 3788 and *P. vivax* = 72) and Gida Ayana (*P. falciparum* = 513, *P. vivax* = 167 and mixed infection of both species = 5) confirmed malaria cases were reported in 2019/2020.

Sherkole and Horazhab health centres are found in the Assosa zone in the Benishangul-Gumuz Region of Ethiopia. Both health centres are located in areas along the western borders of Sudan which has been characterized by the highest transmission intensity [18]. Malaria transmission remains generally high in the western Ethiopian border area near Sudan [19]. Sherkole health centre is located in the Sherkole district which is bordered by Sudan in the north. Sherkole town is about 754 km from Addis Ababa. Horazhab health centre is located in Kurmuk district, and it is bordered by Sudan in the north and west. It is about 769 km from Addis Ababa.

Anger Gute and Warabo health centres are found in Anger Gute town, Gida Ayana district, East Wollega Zone, the Oromia Regional State. Anger Gute town is 360 km away from Addis Ababa. The altitude of the area is between 1200 and 1500 m above sea level.

Malaria transmission in the Anger Gute area is low and stable. *Plasmodium falciparum* infection prevalence among children 2–10 years was  < 5% [18]. Incidence of malaria in and around Anger Gute town was 3.43 per 1000 population at risk of the disease and the malaria trend from 2014 to 2018 indicated nearly unchanged numbers of malaria cases [20].

### Study design

A health facility-based a cross-sectional study was conducted from September to December 2020. Consenting febrile patients seeking malaria diagnosis at the study sites were recruited.

### Sample size

The sample size was determined using a single population formula, using a 13.1% prevalence of malaria in Benishangul-Gumuz regional state [16], a 95% confidence level, and 5% precision. Accordingly, the calculated sample size was 175. With a13% adjustment for non-response rate, a total of 198 uncomplicated falciparum malaria patients were included in the study. The number of patients included from high and low transmission sites was proportional to the number of confirmed *P. falciparum* reported from the study health facilities in 2019 G.C. Accordingly, from a total of 230 samples, 112, 67, and 51 were collected from Sherkole, Gida Ayana and Kurmuc districts, respectively.

### Study population

At each health centre, consenting patients with uncomplicated malaria whose age was greater than 6 months, and microscopically confirmed to be infected with *P. falciparum*, were enrolled. For confirmatory diagnosis of *P. falciparum,* finger-prick blood samples were used for thick and thin blood films for slide microscopy were made. Each slide was stained with 10% Giemsa for 10 min and 100 fields examined before designating a sample negative [21]. Once the patients were microscopically confirmed of *P. falciparum* infection, they were consented to give finger-prick blood samples to prepare dried blood spots (DBS) on filter paper (Whatman No. 1001 320, International Ltd. Maidstone, England). The DBS were kept in plastic bags with desiccants until molecular analysis. Patients with mixed infections with other *Plasmodium* species were excluded from the study.

### Molecular genotyping

Molecular detection of parasite genomic DNA was done at Medical Research Council Unit The Gambia at the London School of Hygiene & Tropical Medicine. DNA extraction was done from DBS using the Chelex protocol as earlier described [22] *Plasmodium falciparum* detection was performed by *var* gene acidic terminal sequence (*varATS*) real-time PCR as previously described [23]. For genetic diversity, the genomic DNA of the parasite was amplified by multiplex primary and nested PCR using allele-specific Primers of *msp1* and *msp*2 as per published protocol [24].

There was one set of primers for the primary PCR (pPCR), which was generic for all 3 allelic families of *msp1*, and 3 sets of primers for the nested PCR (nPCR) that were specific for the allelic families K1, Mad20,and R033 (Table 1). Fluorochrome-labeled forward primers were used for the nPCR to distinguish between the allelic families. Genotypes are distinguished by their fluorescent dye (indicating the allelic family) and by the amplicon size, which was determined by QIAxcel ScreenGel Software, developed for use with the QIAxcel Advanced system.

**Table 1:** Primers, Thermal cycling conditions of the PCR and controls for *msp1* and *msp2*

Gene	PCR round	Primer name	Sequence (5'–3')	Cycling conditions	Positive control
*msp1*	Primary	N1-Fw	GCAGTATTGACAGGTTATGG	Initial denaturation: 94 °C for 5 min; PCR: 30 cycles of 94 °C for 30 s, 45 °C for 45 s, 70 °C for 1.5 min:; final elongation: 70 °C for 10 min	3D7, K1, HB3 and Dd2
		N1-Re	GATTGAAAGGTATTTGAC		
	Nested	K1- Fw	ROX/AATGAAGAAGAAATTACTACAAAAGGTGC	Initial denaturation: 94 °C for 5 min; PCR: 30 cycles of 94 °C for 30 s, 52 °C for 45 s, 70 °C for 1.5 min; final elongation: 70 °C for 10 min	3D7
		K1-Re	GTGTCTTGCTTGCATCAGCTGGAGGGCTTGCACCAG		
		MAD-Fw	6FAM/AAATGAAGGAACAAGTGGAACAGCTGTTAC		Dd2
		MAD-Re	GTGTCTTATCTGAAGGATTTGTACGTCTTGAATTACC		
		RO33-Fw	HEX/TAAAGGATGGAGCAAATACTCAAGTTGTTG		7G8
		RO33-Re	GTGTCTTCAAGTAATTTTGAACTCTATGTTTTAAATC		
*msp2*	Primary	S2-Fw	GAAGGTAATTAAAACATTGTC	Initial denaturation: 94 °C for 5 min; PCR: 30 cycles of 94 °C for 30 s, 45 °C for 45 s, 70 °C for 1.5 min:; final elongation:70 °C for 10 min	3D7, K1 and HB3
		S2-Re	GAGGGATGTTGCTGCTGCTCCACAG		
	Nested	Fc 27- Fw	6FAM/GCATTGCCAGAACTTGAA	Initial denaturation: 94 °C for 5 min; PCR: 30 cycles of 94 °C for 30 s, 52 °C for 45 s, 70 °C for 1.5 min; final elongation: 70 °C for 10 min	Dd2
		3D7-fw	HEX/CTGAAGAGGTACTGGTAGA		3D7
		Stail Re	GTGTCTTGCTTATAATATGAGTATAAGGAGAA		

There was one set of primers for the pPCR, which was generic for the two allelic families of *msp2*, and three primers for the nPCR: one reverse primer that was common for both allelic families and two forward primers that were specific for the allelic families 3D7 and Fc27. Fluorochrome-labelled forward primers are used for the nPCR to distinguish between the allelic families. Genotypes were distinguished by their fluorescent dye (indicating the allelic family) and by the amplicon size which was determined by QIAxcel ScreenGel Software, developed for use with the QIAxcel Advanced system.

### Data analysis

PCR amplicon bands for *msp1* and *msp2* were sized and binned using the QIAxcel ScreenGel Software. SPSS 20.0 statistical software package (SPSS, Inc, Chicago, USA) was used for statistical analysis. The allelic frequency of *msp1* or *msp2* was determined by counting the number of samples observed with a particular allelic variant, divided by the total number of allelic variants observed in all the samples analysed for *msp1* or *msp*2.

An infection with a single allele for both targets was considered as a monoclonal infection and those infections with more than one PCR amplicon for either or both targets were considered polyclonal. Mean MOI in both settings of malaria transmission was determined as the ratio of the total number of amplified bands detected in *msp1* and *msp2* by the number of samples positive for *msp1* and *msp2*. Frequencies of *msp1* or *msp2* alleles among the study sites were analysed using the chi-square test. Binary logistic regression analysis was done to determine factors associated with the prevalence and multiplicity of *P. falciparum* infection. In addition, the student’s t-test was used to compare the mean MOI of both loci between high and low transmission sites.

Expected heterozygosity (HE), a measure of genetic diversity, was calculated by using the following formula: HE = [n/(n − 1)][(1 − Σpi2)], where n is the number of isolates sampled and pi is the allele frequency at a given locus. A p-value of less or equal to 0.05 was considered suggestive of a statistically significant difference between sites.

### Ethical considerations

Ethical clearance was obtained from the Ethiopian National Ethics Review Committee and Addis Ababa University, Aklilu Lemma Institute of Pathobiology, IRB. Permission to conduct the study at the health facilities was sought from the relevant regional and district health authorities. Written informed consent was obtained from adult study participants and a parent or guardian of a child. Written informed assent was also taken from children.

## Results

Out of 230 microscopically confirmed *P. falciparum* cases enrolled in the study, 225 of them tested positive by PCR. 72.4% (163/225) and 27.6% (62/225) PCR positive samples were collected from high and low malaria transmission sites, respectively. The mean age of study participants was 17.8 ± 12.7 years and with an age range of 7 months–75 years old. The ratio of males to females was 1.27: 1. The prevalence of falciparum malaria was higher in children less than 15 years as compared with those  ≥ 15 years old (AOR = 2.438, P = 0.005). No significant difference in *P. falciparum* infections in gender was observed (p > 0.05), (Table 2).

**Table 2:** Sociodemographic characteristics and occurrence of malaria in western Ethiopia

Variables	High transmission	Low transmission	COR	P-value	AOR	P-value	High transmission
Sex							
Male	85	41	1.79	0.061	1.827	0.057	85
Female	78	21	1		1		78
Age categories							
< 15 years	90	21	2.41	0.005	2.438	0.005	90
≥ 15 years	73	41	1		1		73

*COR* Crude Odds Ratio, *AOR* Adjusted Odds Ratio

### Genetic polymorphism of msp1 and msp2 genes

*Msp1* genotyping was successful in 87% (141/163) and 88.7% (55/62) of samples collected from high and low transmission areas, respectively. Similarly, for *msp2* genotyping, 85.3% (139/163) and 96.8% (60/62) samples were successfully genotyped from high and low transmission sites, respectively. The three allelic families of *msp1* (K1, MAD20, and RO33) and FC27 and 3D7 of *msp*2 allelic families were identified in all study sites. There was no difference in the frequency of *msp1* or *msp2* allelic families by study sites (P > 0.05) (Table 3).

**Table 3:** Allelic frequency of *msp1* and *msp2* genotypes in study areas

Genes and alleles	Sherkole	Kurmuc	Anger Gute	p-value
*Msp1* MAD20	70	28	44	0.337
K1	13	2	2	
RO 33	1	2	2	
MAD20 + K1	16	5	6	
MAD 20 + RO33	2	2	1	
*Msp2* FC27	92	40	57	0.99
3D7	2	1	1	
FC27 + 3D7	3	1	2	

**Fig. 2:**
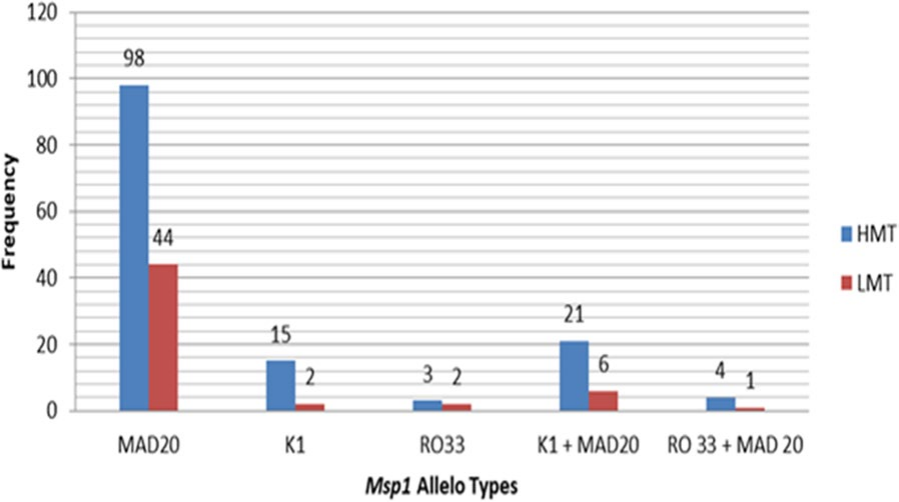
Distribution of *msp1* allele types among *P. falciparum* isolates in different levels of malaria transmission, western Ethiopia. *HMT* high malaria transmission, *LMT* low malaria transmission

For *msp*1, the MAD20 allele was dominant; with a total of 87.2% (123/141), 25.5% (36/141), and 5% (7/141) of the samples from high transmission sites had MAD20, K1, and RO33 allelic families of *msp*1, respectively. Similarly, 92.7% (51/55), 14.5% (8/55), and 5.45% (3/55) samples collected from low malaria transmission settings had MAD20, K1, and RO33 *msp*1 allelic families, respectively. Multiclonal infections with K1 + MAD20, and MAD20 + R033 combinations were also observed. In the high transmission setting, K1 + MAD20 accounted for 14.9% (21/141) while the frequency of this allelic combination was 10.9%(6/55) in the low transmission setting (Fig. 2). Allelic combinations of K1 and RO33, and K1/MAD20/RO33 were not detected in any infection.

The fragment size of MAD 20 alleles observed in high malaria transmission area (103–308 bp) was similar to that seen in *P. falciparum* isolates from low malaria transmission site (103–295 bp). A similar pattern was also observed with the K1 alleles, where sizes in high malaria transmission ranged from 167 to 282 bp while those from low malaria transmission sites ranged from 167 to 269 bp. In addition, a similar pattern was observed in RO33 where *P. falciparum* isolates in high and low malaria transmission areas had fragment sizes of (160–162 bp) and (164–165 bp), respectively.

**Fig. 3:**
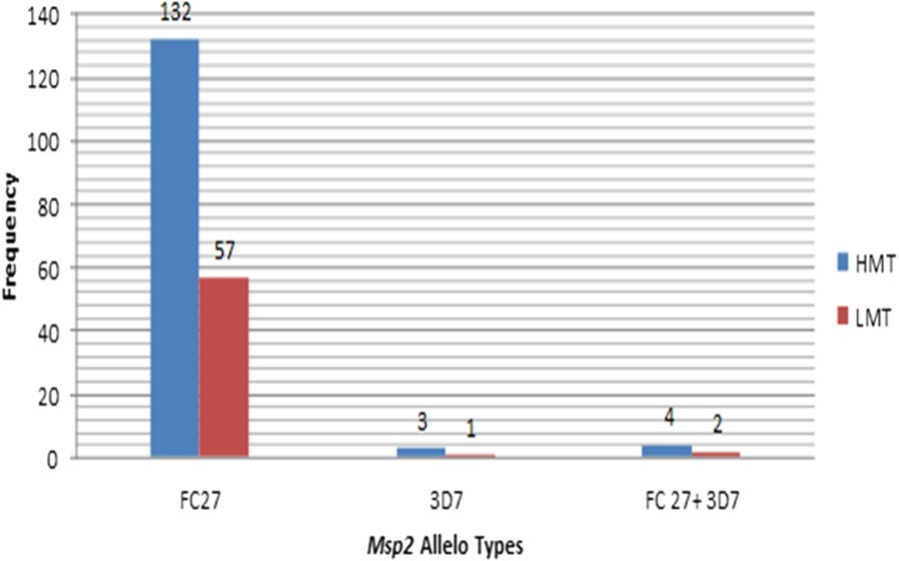
Distribution of *msp*2 Allelo types by the intensity of malaria transmission in western Ethiopia

Both FC27 and 3D7 *msp2* allelic types identified among the samples collected from both study sites. However; FC27 was the frequent allelic type in western Ethiopia. There was no difference in the frequency of this allele in high transmission 97.8% (136/139) and low transmission 98.3% (159/160) areas. Few multi-allelic infections combining 3D7 + FC27 families were detected (Fig. 3). FC27 fragment size ranging from (159–787 bp) and (144–488 bp) were identified among the isolates collected from high and low malaria transmission study sites, respectively. Similar pattern was also observed with the 3D7 alleles, where sizes in high malaria transmission areas ranged from 270 to 285 bp while those from low malaria transmission ranged from 276 to 288 bp.

In western Ethiopia, monoclonal *P. falciparum* infections were predominant, representing 82.3% (116/141) and 87.3% (48/55) of the samples genotyped for *msp1* in high and low transmission sites, respectively. Similarly, 97.2% (135/139) and 96.7% (58/60) of isolates from high and low transmission areas were monoclonal *msp2,* respectively. No significant difference was observed in the proportion of *msp1* or *msp2* multiplicity of infection with sex, and age group of study participants and intensity of transmission (P > 0.05) (Table 4).

**Table 4:** Proportion of multiplicity of *P. falciparum* infection in relation to sociodemographic indices of patients and level of malaria transmission, western Ethiopia

Variables	*msp1 *(n = 196)		AOR	P-value	*msp2 *(n = 199)		AOR	P-value
	Monoclonal	Polyclonal			Monoclonal	Polyclonal		
Sex								
Male	95	15	0.67	0.31	107	4	1.61	0.6
Female	69	17			86	2		
Age categories								
< 15 years	82	15	1.2	0.64	134	3	0.95	0.93
≥ 15 years	82	17	1		58	3	1	
Level of transmission								
High	116	25	0.69	0.43	135	4	1.11	0.91
Low	48	7	1		58	2	1	

*Plasmodium falciparum* isolates collected from western Ethiopia had low mean MOI and there was no significant difference in mean MOI between high transmission (mean MOI = 1.104) and low transmission (mean MOI = 1.08) (p > 0.05). The expected heterozygosity of the *msp1* gene was slightly higher in high malaria intensity sites (He = 0.17) than isolates from low malaria intensity (He = 0.12). On the other hand, heterozygosity of the *msp2* gene was not different in both settings (Pf*msp2*: 0.04; pf*msp2*: 0.03), (Table 5).

**Table 5:** MOI and heterozygosity of *msp*1 and *msp*2 of *P. falciparum* in western Ethiopia

Gene/genes	high transmission (n = 280)	Low transmission (n = 115)	P- value
mean MOI	1.104	1.08	0.44
*msp*1 He	0.17	0.12	
*msp*2 He	0.04	0.03	

## Discussion

Measuring the complexity of infection and genetic diversity of parasite populations across different endemicities may be used as an indicator to evaluate the efficacy of ongoing control and elimination strategies. While a few studies have investigated MOI and genetic diversity of the *P. falciparum* population circulating in western Ethiopia, these didn’t represent populations with relatively high and low transmission sites as presented here. The prevalence of uncomplicated falciparum malaria in the populations studied was higher in children  ≤ 15 years than those  ≥ 15 years old (AOR = 2.438, P = 0.005) and this was in agreement with previous reports from Ethiopia and Kenya [25, 26]. Immunity to malaria by an individual depends on their age, the number of infectious mosquito bites [27], and the intensity of transmission [28, 29]. In high transmission areas, the burden of malaria is mainly among young children unlike in areas with low transmission, where the population has the low exposure to infection and malaria occurs in all age groups [^25^, ^30^, ^31^].

The three allelic families of *msp1* (K1, MAD20, and RO33) and FC27 and 3D7 of *msp*2 allelic families were identified in the study areas. However, MAD 20 of *msp1* and FC27 of *msp2* allelic families were the predominant alleles in both areas with variable malaria endemicity. There was no difference in the frequency of *msp1* or *msp2* allelic families by the intensity of transmission. Therefore, the population composition of *P. falciparum* isolates in western Ethiopia shows monoclonality infections regardless of the difference in intensity of transmission intensity and geographic separation of the parasites. This finding suggests the likely presence of a high level of inbreeding among the circulating clones between the study sites. Previous reports had also indicated that malaria parasites in Ethiopia have moderate levels of genetic diversity and a similar population structure of the parasite [32]. They presented the lowest levels of heterozygosity in a continent-wide *P. falciparum* genomic analysis [33], indicating the need to further determine how they have evolved and are responding to the general interventions recommended for all malaria populations.

The predominance of the MAD 20 for *msp1* and the FC27 allelic family for *msp2* among *P. falciparum* isolates was consistent with previous studies done in countries; Myanmar [34], Vietnam  [35], Indonesia [36], that showed MAD 20 predominance and Pakistan [37, 38], and Malaysia [39] that reported FC27 of *msp2* dominance; The predominance of the MAD 20 for *msp1* and the FC27 allelic family for *msp2* were reported in other parts of Africa; North Central Nigeria [40], Equatorial Guinea [41], and Northwest Ethiopia [42]. Although this corroborates previous studies from Ethiopia finding similar patterns with the predominance of MAD 20 [43] and FC27 [44, 45]. Predominance of MAD 20 of *msp1* and 3D7/IC1 of *msp2* in other reports had shown the predominance of K1 of *msp1* and 3D7/IC1 of *msp2* in southwestern Ethiopia [46]. This is either due to variance between populations or changes that may have occurred over time following selection by pressure from interventions and reducing transmission intensity.

Most of the participants had monoclonal infections at both *msp1* and *msp2* loci in both settings*.* As with previous reports, the multiplicity of infection (MOI) was also not associated with age groups [40, 44]. This was in contrast to other studies that found a correlation between polyclonal infections with age groups [47], though this was in a high transmission region in Burkina-Faso in West Africa. MOI was not associated with the sex of the patients as reported from Burkina Faso[47]. Overall, *P. falciparum* isolates from Western Ethiopia showed low mean MOI and limited genetic diversity, with no differences based on the intensity of transmission, a pattern that has been seen in some malaria-endemic regions of Africa, the prevalence of infections have generally become low and imported cases are common [15, 48, 49]. Examples of these include Senegal and Eswatini, which are now heading towards pre-elimination. Though high MOI and genetic diversity have been associated with higher malaria-endemicity, these indices as determined by *msp1* and *msp2* typing may not be sensitive enough at a lower overall transmission level as seen in Ethiopia [^12^, ^41^, ^50^, ^51^]. However, the lack of differences with the relative variance in transmission intensities might be an indication that malaria control measures that reduce entomological inoculation rate [52, 53], and deployment of artemisinin-combination therapy that reduce population diversity by removing the drug-sensitive parasites [54] have been effective in Ethiopia. It is also possible differences in human demography, ecology, and *Anopheles* mosquito vector might be shaping *P. falciparum* population structure in malaria- endemic sites [55–57]. Within-host competitive interactions of *P. falciparum* may also determine the diversity of the parasite [3]. Malaria transmission in Ethiopia is driven mainly by *Anopheles arabiensis*, while *Anopheles pharoensis*, *Anopheles funestus* and *Anopheles nili* are secondary vectors [58, 59], unlike the situation across the most endemic regions of west and central Africa, where *Anopheles gambiae* is mostly seen.

## Conclusion

*Plasmodium falciparum* isolates were mainly monoclonal with low MOI and lesser genetic diversity. Mean MOI and diversity of the parasite were not associated with the intensity of malaria transmission. Hence, MOI and the genetic diversity of *P. falciparum* might not be a good predictor of transmission intensity in all malaria- endemic areas. There is a need to find out an inclusive measurement of the intensity of transmission in all malaria-endemic areas. The observation of low genetic diversity and MOI in the study area may signal the effectiveness of the recent deployment of massive malaria control strategies in Ethiopia; however, this needs further studies to map the different intervention strategies and other parameters that could affect parasite genetic diversity.

## Data Availability

The datasets and analysed results of the study are available from the corresponding author and can be obtained on reasonable request.

## Abbreviations

DBS: Dried blood spots
IRB: Institute Review Board
PCR: Polymerase chain reaction
*varATS*: Var gene acidic terminal sequence
MOI: Multiplicity of infection
*msp1*: Merozoite surface protein 1
*msp2*: Merozoite surface protein
